# Bipolar disorder, pregnancy, COVID-19: Electroconvulsive therapy is needed!

**DOI:** 10.1192/j.eurpsy.2021.1648

**Published:** 2021-08-13

**Authors:** M.F. Tascon Guerra, M.V. López Rodrigo, G. Manrique Ovejero

**Affiliations:** 1 Psychiatry, Hospital Ntra Sra del Prado, Talavera de la Reina, Toledo, Spain; 2 Family Medicine, Hospital Ntra Sra del Prado, Talavera de la Reina, Toledo, Spain

**Keywords:** pregnancy, Electroconvulsive therapy, bipolar disorder, COVID-19

## Abstract

**Introduction:**

Treating pregnant women with bipolar disorder is among the most challenging clinical endeavors. Patients and clinicians are faced with difficult choices at every turn, and no approach is without risk. Many primary mood stabilizers have been associated with risk of congenital malformations. In the last 15 years, there has been an increase of antepartum use of atypical antipsychotic drugs, many of which could be viable alternatives to mood stabilizers. Electroconvulsive therapy has been recommended as a safe and efficacious treatment of bipolar depressive and manic episodes in pregnant women.

**Objectives:**

This case presents a 24-year-old woman, with COVID-19 infection, that underwent an acute manic episode at her 20-weeks-pregnancy. The goal was to stabilize the patient by the use of electroconvulsive therapy.

**Methods:**

The patient was admitted in isolation in the psychiatric ward. Treatment was started with olanzapine 20mg/d and lorazepam 4mg/d. The patient maintained psychotic agitation that required higher dosage, while on the second week of isolation the PCR test was negative. After six weeks of treatment severe manic symptoms continued and electroconvulsive therapy was started.

**Results:**

She received 10 electroconvulsive therapy sessions. The patient showed a substantial clinical improvement after the seventh administration. She gave birth at 37 weeks, with no complications during labor (Apgar 9/10).

**Conclusions:**

Electroconvulsive therapy has been shown as a suitable option for patients with severe psychiatric disorders in the pregnancy period, either medication resistant illness and psychotic agitation.

**Disclosure:**

No significant relationships.

